# Replication Protein A (RPA) Mediates Radio-Resistance of Glioblastoma Cancer Stem-Like Cells

**DOI:** 10.3390/ijms21051588

**Published:** 2020-02-26

**Authors:** Henriette Pedersen, Elisabeth Anne Adanma Obara, Kirstine Juul Elbæk, Kristoffer Vitting-Serup, Petra Hamerlik

**Affiliations:** 1 Brain Tumor Biology, Danish Cancer Society Research Center, Strandboulevarden 49, Copenhagen DK-2100, Denmark; henped@cancer.dk (H.P.); elioba92@gmail.com (E.A.A.O.); kijuel@cancer.dk (K.J.E.); kvs@cancer.dk (K.V.-S.); 2 Biotech Research and Innovation Centre & Department of Computational and RNA Biology, Department of Biology, Faculty of Health and Medical Sciences, University of Copenhagen, Ole Maaløes Vej 5, Copenhagen 2200-DK, Denmark; 3 Department of Drug Design and Pharmacology, Copenhagen University, Jagtvej 160, Copenhagen 2100-DK, Denmark

**Keywords:** glioblastoma, cancer stem-like cells, RPA, DNA damage, radio-resistance

## Abstract

Glioblastoma (GBM) is among the deadliest of solid tumors with median survival rates of approximately 12–15 months despite maximal therapeutic intervention. A rare population of self-renewing cells referred to as GBM cancer stem-like cells (GSCs) are believed to be the source of inevitable recurrence in GBM. GSCs exhibit preferential activation of the DNA damage response pathway (DDR) and evade ionizing radiation (IR) therapy by superior execution of DNA repair compared to their differentiated counterparts, differentiated GBM cells (DGCs). Replication Protein A (RPA) plays a central role in most of the DNA metabolic processes essential for genomic stability, including DNA repair. Here, we show that RPA is preferentially expressed by GSCs and high RPA expression informs poor glioma patient survival. RPA loss either by shRNA-mediated silencing or chemical inhibition impairs GSCs’ survival and self-renewal and most importantly, sensitizes these cells to IR. This newly uncovered role of RPA in GSCs supports its potential clinical significance as a druggable biomarker in GBM.

## 1. Introduction

A key feature that distinquishes cancer cells from most normal cells is their sustained proliferation. Faithful replication and segregation of DNA material into daugther cells is therefore fundamental to genome stability and cell survival. Seminal studies have shown that pre-cancerous lesions display baseline DNA damage, supposedly caused by unscheduled replication initiation upon oncogenic stimuli [1]. Replication stress (RS) occurs when replication forks encounter aberrant DNA structures, which either stall, block or prematurely terminate their progression, ultimately causing fork collapse and the formation of long stretches of single-stranded DNA (ssDNA) [1,2]. ssDNA is bound with replication protein A (RPA), a heterotrimeric protein composed of three subunits, RPA14, RPA32 and RPA70. Although a correlation between RS and genomic instability is well acknowledged, molecular mechanisms underpinning this coupling remain elusive [2,3,4]. Glioblastoma (GBM, a World Health Organization/WHO grade IV glioma) is among the deadliest of solid tumors with median survival rates of approximately 12–15 months despite maximal therapeutic intervention [5]. Rampant genomic instability and radio-resistance are among the hallmarks of GBM. A population of theraputically resistant self-renewing cancer stem-like cells (CSCs) stands at the apex of celullar hierarchies found in these aggressive heterogenous tumors. In GBM, the CSCs (GBM cancer stem-like cells; GSCs), prospectively isolated based on the expression of cell surface makers such as CD133, exhibit superior DNA repair capacity, which protect them from the impact of genotoxic therapies such as ionizing radiation (IR) or chemotherapy by alkylating agent temozolomide [6,7,8,9]. IR, a standard of care for GBM, induces a plethora of DNA lesions, including oxidative base damages, ssDNA breaks and double-strand breaks (DSBs). In response to such DNA damage, a network of events collectively termed as the DNA damage response pathway (DDR) is activated and includes DNA damage recognition, activation of check-points, cell cycle arrest and ultimately cell death [2,10]. DDR is primarily coordinated by two signaling cascades: ataxia telaniectasia mutated (ATM)-checkpoint kinase 2 (CHK2) pathway and ataxia telengiectasia and Rad3-related (ATR)-checkpoint kinase 1 (CHK1) pathway, of which the activation then results in the phosphorylation of H2AX at Ser139 (H2AX), a marker for the double-strand DNA breaks (DSBs) [11]. Upon DNA damage or RS, the activation of these pathways leads to hyperphosphorylation of RPA, which modulates its role in response to various genotoxic insults [3].

A central challenge of IR therapy is to maximize the cancer cell eradication while minimizing the impact on healthy tissues. Thus, targeting factors dispensable for normal but essential for cancer cells to evade the consequences of genotoxic insults may help us to enhance the efficacy of IR whilst minimizing side effects on normal brain parenchyma [12]. In this study, we show that RPA is preferentially expressed by the radio-resistant pool of GSCs. RPA loss impairs the survival and self-renewing potential of GSCs while driving them into cell death by apoptosis. Most importantly, both silencing by shRNA and chemical inhibition of RPA by (1Z)-1-[(2-Hydroxyanilino)methylidene]naphthalen-2-one (HAMNO) induce DSBs, a challenge that impairs the DNA repair capacity of GSCs, ultimately increasing their sensitivity to IR.

## 2. Results

### 2.1. RPA is Overexpressed in High-Grade Gliomas and Informs Patient Survival

**Figure 1:**
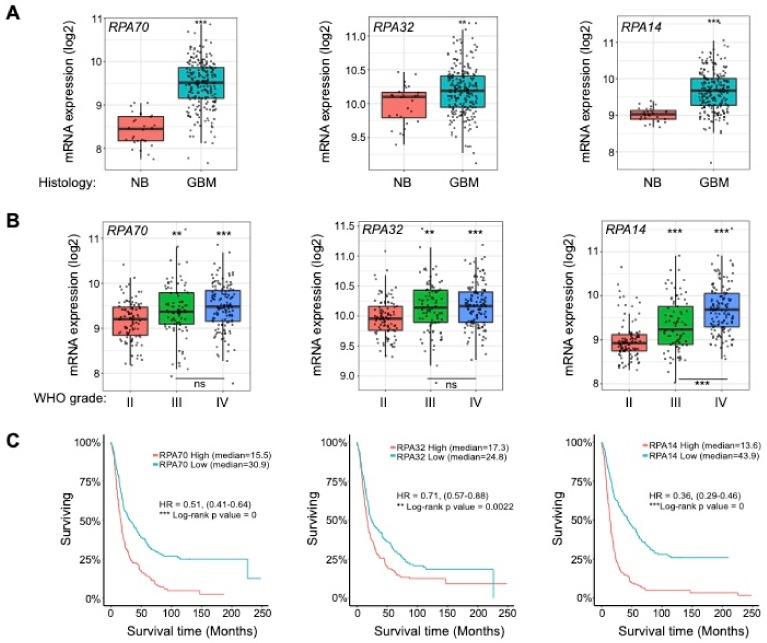
Replication protein A (*RPA*) is overexpressed in high-grade gliomas and informs patient survival. (**A**) *RPA70*, *RPA32*, *RPA14* expression analysis of REMBRANDT data (the National Cancer Institute’s repository) comparing glioblastoma (GBM) and normal brain (NB) controls. (**B**) *RPA70*, *RPA32*, *RPA14* expression analysis of REMBRANDT data (the National Cancer Institute’s repository) comparing WHO grade II, III and IV gliomas. Statistical significance was tested using Tukey’s honestly significant difference test, HSD. ns: not significant; ** *p* < 0.01; *** *p* < 0.001. (**C**) Kaplan-Meier survival analysis of REMBRANDT glioma data set shows that high *RPA* expression (all subunits) informs poor patient prognosis.

Previous studies have implied that RS is an early event in the formation of GBM [13]. Since the heterotrimeric protein RPA is among the first responders to RS and by its interaction with ATR mediates the activation of DDR [3], we sought to examine its expression in a cohort of GBM patients and compared it to that of heatlhy controls. We chose the publicaly available REMBRANDT and The Cancer Genome Atlas (TCGA) glioma data sets and interrogated the expression of all three *RPA* subunits, *RPA70*, *RPA32* and *RPA14*, using the gliovis platform (http://gliovis.bioinfo.cnio.es/,visited on 12/02/2020) [14]. As shown in Figure 1A, Supplementary Figure S1A, all three *RPA* subunits *RPA70*, *RPA32* and *RPA14* were expressed at higher levels in GBM compared to normal brain (NB) controls. Moreover, *RPA* was overexpressed in high-grade gliomas (Word Health Organization, WHO grade III and IV) compared to low grade lesions (WHO grade II) (Figure 1B, Supplementary Figure S1B). The Kaplan-Meier survival analysis revealed that low *RPA70*, *RPA32* and *RPA14* expression associates with a better prognosis of glioma patients (Figure 1C, Supplementary Figure S1C). When assessing the impact of *RPA* expression on the survival of GBM patients only, Kaplan-Meier survival analysis showed that high expresion of *RPA70* and *RPA14*, but not *RPA32,* informs worse patient survival (Supplementary Figure S2A). A multivariate Cox proportional hazard regression analysis of the TCGA data sets (see Supplementary Figure S2B and Supplementary Tables S1 and S2) showed that only *RPA14* expression in low-grade gliomas could serve as an independent prognostic factor. The prognostic value of *RPA70* and *RPA32* expression is dependent on other prognostic factors such as WHO grade, age and isocitrate dehydrogenase (IDH) status in both low- and high-grade gliomas (see Supplementary Figure S2B and Supplementary Tables S1 and S2).

### 2.2. RPA Expression is Crucial for the Maintenance of Glioblastoma Cancer Stem-Like Cells

**Figure 2:**
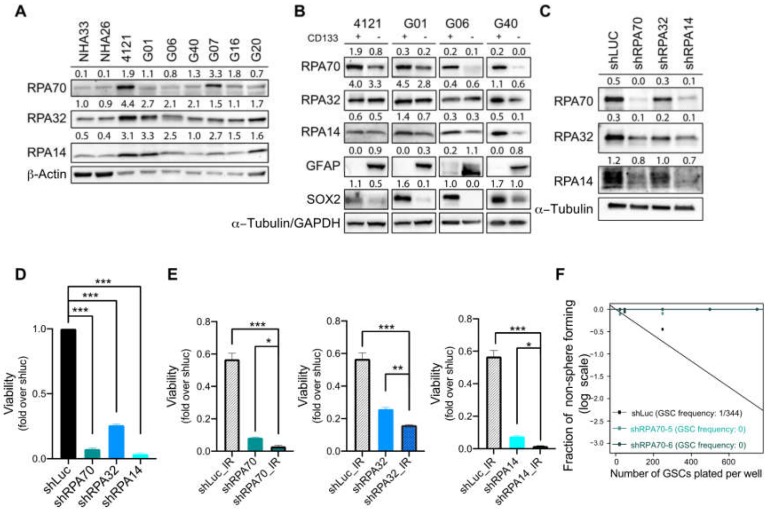
RPA expression is crucial for the maintenance of glioblastoma cancer stem-like cells. (**A**) Immunoblot analysis of RPA70, RPA32 and RPA14 in a panel of GBM primary cell lines (4121, G01, G06, G40, G07, G16 and G20) and two normal human astrocyte cell lines (NHA33, NHA26). β-Actin was used as a loading control. (**B**) Immunoblot analysis of RPA70, RPA32 and RPA14 expression in matched pairs of glioblastoma cancer stem-like cells (GSCs) and differentiated GBM cells (DGCs) isolated from 4121, G01, G06 and G40 primary GBM cell lines. α-Tubulin or Glyceraldehyde 3-phosphate dehydrogenase (GAPDH) was used as a loading control. (**C**) Immunoblot analysis validating knockdown efficiencies of lentiviral shRNAs targeting RPA70, RPA32 and RPA14 in G01-GSCs. α-Tubulin was used as a loading control. (**D**) CellTiter-Glo luminescent viability assay (Promega) of G01-GSCs transduced with lentiviral shRNA targeting RPA70, RPA32 or RPA14 assessed 72 h after virus wash. Data are presented as mean ± SD. Statistical significance was tested using a two-way ANOVA with post-hoc Sidak’s multiple comparisons test, where *** *p* < 0.001. *N* = 3. (**E**) CellTiter-Glo luminescent viability assay (Promega) of G01-GSCs transduced with lentiviral shRNA targeting RPA70, RPA32 or RPA14 alone or 24 h after irradiation (3 Gy; COMBO) or sham-irradiated and assessed for viability 72 h later. (**E**) Extreme limiting dilution assay (ELDA) of G01-GSCs transduced with lentiviral shRNA targeting of RPA70 (2 independent shRNAs) shows significantly impaired self-renewal of G01-GSCs after 10 days. Data are presented as mean ± SD. Statistical significance was tested using a two-way ANOVA with post-hoc Sidak’s multiple comparisons test, where * *p* < 0.05, ** *p* < 0.01, *** *p* < 0.001. *N* = 3.

Our previous work has shown that gliomas, in general, and GSCs, in particular, exhibit high reactive oxygen species (ROS) production and with that associated high baseline of oxidative DNA damage, which leads to the accumulation of ssDNA [13,14,15]. Since RPA coats ssDNA immediately upon its inception, we sought to investigate the RPA protein expression in patient-derived primary cell cultures passed as mouse xenografts. On immunoblot, all three RPA subunits were expressed at higher levels in our collection of primary GBM cell lines compared to normal human astrocytes (NHA33 and NHA26; Figure 2A). Next, we assessed the RPA expression in acutely dissociated and Magnetic-Activated Cell Sorting (MACS) -sorted matched-paired GSCs (CD133 positive) and differentiated GBM cells (DGCs; CD133 negative) from the 4121, G01, G06 and G40 lines, and found RPA subunits RPA70 and RPA14 were preferentially expressed by GSCs (Figure 2B). To further interrogate the role of RPA, we silenced RPA using subunit-specific lentiviral shRNAs (shRPA70, shRPA32, shRPA14) in GSCs isolated from the G01 line (further denoted as G01-GSCs). Immunoblot analysis revealed that silencing of any of the individual subunits negatively impacts the expression of the other two remaining subunits (Figure 2C), suggesting that targeting of just one of the subunits is sufficient for abrogating the overall function of total RPA. Lentivirus-mediated knockdown of RPA subunits impaired the viability of G01-GSCs as measured by CellTiter-Glo luminiscence cell viability assay (Figure 2D). Most importantly, RPA silencing sensitized G01-GSCs to IR (Figure 2E) and reduced their capacity to self-renew (Figure 2F), thereby supporting our hypothesis that RPA mediates the radio-resistant phenotype of this aggressive cell population and supported the notion that a successful eradication of RPA function may impair their capacity to evade radio-therapy.

### 2.3. RPA70-Specific Inhibitor HAMNO Targets Glioblastoma Cancer Stem-Like Cells

**Figure 3:**
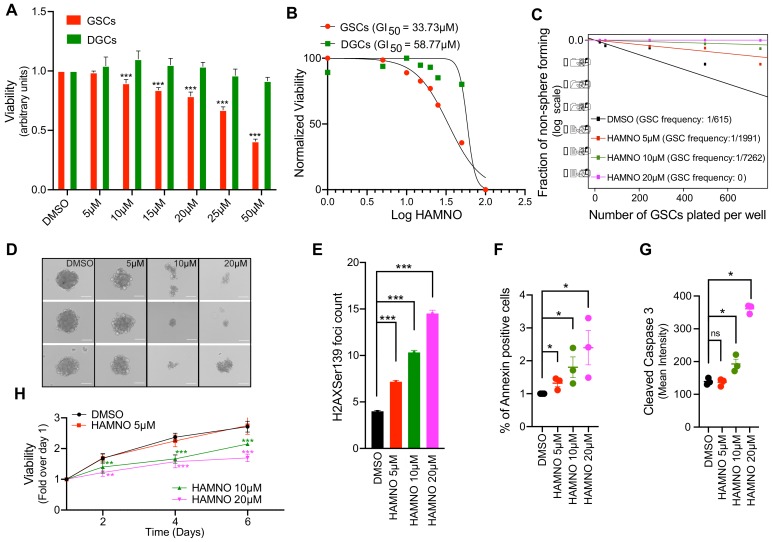
RPA70-specific inhibitor 2-hydroxyanilino),methylidene]naphtalen-2-one (HAMNO) targets glioblastoma cancer stem-like cells. (**A**) CellTiter-Glo luminescent viability assay (Promega) of matched GSCs and DGCs treated with vehicle control (DMSO) or increasing doses of HAMNO—5, 10, 15, 20, 25, 50 and 100 0μM assessed at 72 h post treatment. (**B**) A graph showing HAMNO GI_50_ (72 h) in matched GSCs and differentiated GBM cells (DGCs) assessed by CellTiter-Glo luminescent viability assay (Promega). (**C**) Extreme limiting dilution assay (ELDA) in the presence of vehicle control (DMSO) or increasing doses of HAMNO—5, 10 and 20 μM shows significantly impaired self-renewal of GSCs 10 days after treatment. (**D**) Representative images of neurospheres (from three independent experiments, day 10 post-plating) formed in the presence of vehicle control (DMSO) or increasing doses of HAMNO—5, 10 and 20 μM. (**E**) Microscopy-based quantification of γH2AX foci count in GSCs after exposure (48 h) to increasing doses of HAMNO—5, 10 and 20 μM. Data are presented as mean ± SD. Statistical significance was tested using one-way ANOVA, where *** *p* < 0.001. (**F**) Apoptosis was assessed by Fluorescence-Activated Cell Sorting (FACS) analysis of annexin V-positive cells. The graph represents data from 3 independent experiments, where GSCs where treated for 36 h with increasing doses of HAMNO—5, 10 and 20 μM or vehicle control (DMSO). (**G**) Apoptosis was assessed by FACS analysis of cleaved caspase-3 -positive cells. The graph represents data from three independent experiments, where GSCs where treated for 24 h with increasing doses of HAMNO—5, 10 and 20 μM or vehicle control (DMSO). Data are presented as mean ± SD. *N* = 3. Statistical significance was tested using Mann–Whitney Test, where * *p* < 0.05. (**H**) Viability of GSCs treated with vehicle control (DMSO) or increasing doses of HAMNO—5, 10 and 20 μM assessed by the IncuCyte live cell analysis system over a period of six days. Data are presented as mean ± SD. Statistical significance was tested using a two-way ANOVA with post-hoc Sidak’s multiple comparisons test, where ** *p* < 0.01, *** *p* < 0.001. *N* = 3.

RPA is involved in almost all crucial aspects of DNA metabolism and exerts its functions through interaction with DNA as well as other proteins implicated in these processes, including ATR, Rad51, BRCA1/2, p53 etc. [16,17]. HAMNO is a small molecular inhibitor that targets the interaction domain of RPA70 and prevents its binding to ATR, which is indispensable for authophosphorylation events leading to DDR activation under RS conditions [18]. First, we sought to examine whether differential expression of RPA in matched G01-GSCs and G01-DGCs correlates with their sensitivity to HAMNO. Indeed, G01-GSCs showed superior sensitivity to HAMNO in a dose-dependent manner (Figure 3A). The HAMNO GI_50_ of G01-GSCs (33.73 μM) was nearly half of that measure for G01-DGCs (58.77 μM) (Figure 3B). Furthermore, the exposure to HAMNO compromised the self-renewing capacity of GSCs, as evidenced by decreased size and frequency of neurospheres formed (Figure 3C–D).

To assess the impact of RPA inhibition on DSB formation using microscopy-based assay, we have scored γH2AX foci accumulation after treating G01-GSCs with increasing concentrations of HAMNO (Figure 3E). RPA inhibition in this experiment resulted in a dose-dependent accumulation of DSBs, a phenomenon that correlated with an increased death of G01-GSCs by apoptosis as assessed by annexin V and cleaved caspase-3 staining (Figure 3F–G). While 5 μM HAMNO induced significant damage, apoptosis and negatively impacted the sphere formation, only concentrations of 10 and 20 μM caused a significant decrease int G01-GSCs’ viability measured by CellTiterGlo luminiscent assay over a perior of six days (Figure 3H).

### 2.4. HAMNO Treatment Sensitizes Glioblastoma Cancer Stem-Like Cells to Ionizing Radiation

**Figure 4:**
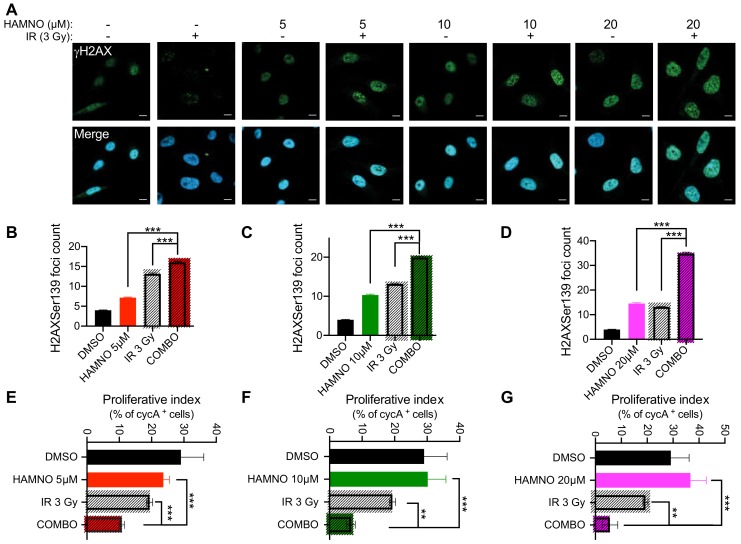
HAMNO treatment induces DNA damage and activates cell cycle checkpoints in glioblastoma cancer stem-like cells. (**A**) Representative confocal microscopy images of γH2AX foci in G01-GSCs treated for 24 h with a vehicle control (DMSO) or increasing doses of HAMNO—5, 10 and 20 μM; then irradiated (COMBO) or sham-irradiated and incubated for an additional 24 h. (**B**–**D**) Microscopy-based quantification of γH2AX foci in G01-GSCs treated for 24 h with a vehicle control (DMSO) or increasing doses of HAMNO—5, 10 and 20 μM; then irradiated (COMBO) or sham-irradiated and incubated for additional 24 h. (**E**–**G**) Microscopy-based quantification of a proliferative index (% of nuclear cyclin A-positive cells) in G01-GSCs treated for 24 h with a vehicle control (DMSO) or increasing doses of HAMNO—5, 10 and 20 μM; then irradiated (COMBO) or sham-irradiated and incubated for additional 24 h. Data are presented as mean ± SD. Statistical significance was tested using one-way ANOVA, where ** *p* < 0.01, *** *p* < 0.001. *N* = 3.

**Figure 5:**
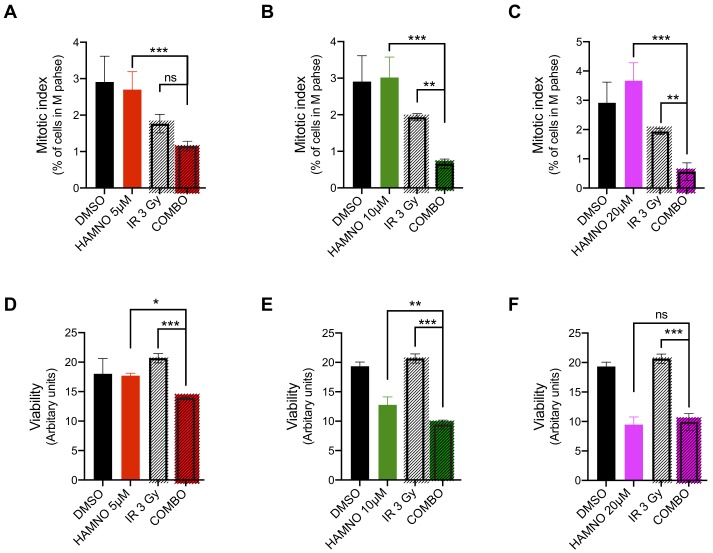
HAMNO treatment lowers the mitotic index and sensitizes glioblastoma cancer stem-like cells to ionizing radiation. (**A**–**B**) Microscopy-based quantification of a mitotic index (% of cells in mitosis) in G01-GSCs treated for 24 h with a vehicle control (DMSO) or increasing doses of HAMNO—5, 10 and 20 μM; then irradiated (COMBO) or sham-irradiated and incubated for additional 24 h. Data are presented as mean ± SD. Statistical significance was tested using one-way ANOVA test, where * *p* < 0.05, ** *p* < 0.01, *** *p* < 0.001; ns = not significant. *N* = 3. (**D**–**E**) CellTiter-Glo luminescent viability assay (Promega) of G01-GSCs treated for 24 h with a vehicle control (DMSO) or increasing doses of HAMNO—5, 10 and 20 μM; then irradiated (COMBO) or sham-irradiated and incubated for additional 48 h. Data are presented as mean ± SD. Statistical significance was tested using a two-way ANOVA with post-hoc Sidak’s multiple comparisons test, where * *p* < 0.01, ** *p* < 0.01, *** *p* < 0.001. ns = not significant. *N* = 3.

GSCs evade DNA damaging therapies by preferential DDR activation [6,9,19]. We observed that HAMNO treatment alone induced DSBs in GSCs, resulting in apoptosis. Thus, we hypothesized that such a challenge can sensitize these aggressive cells to IR. Upon exposure to HAMNO (24 h) or a vehicle control (DMSO), we irradiated G01-GSCs with 3 Gy or sham-irradiated and measured γH2AX foci counts 24 h later. The combination of HAMNO and IR was more effective at inducing DNA damage (irrespective of dose) compared to either of the monotherapies alone (Figure 4A–D). An increase in DNA damage led to cell cycle checkpoint activation, as evidenced by reduced proliferative (% of actively proliferating S phase cells; Figure 4E–G) and mitotic indexes (% of mitotic cells; Figure 5A–C). This combinational impact on cell cycle checkpoints translated into significant decrease in cell survival, where G01-GSCs treated with IR or HAMNO (5 or 10 μM) exhibited better survival than those exposed to their combination (Figure 5D–F).

## 3. Discussion

Single-stranded DNA (ssDNA) is perhaps one of the most ubiquitous and important biological intermediates formed throughout the life of cells [20,21]. Genomes of cells are under continous attack by exogenous and endogenous factors resulting in DNA damage, which, if left unrepaired, leads to genomic instability. RPA, a heterotrimeric ssDNA-binding protein, is required for each of the three major DNA repair pathways: nucleotide excision repair (NER), base excision repair (BER), and DSBs repair [3,4]. Due to its extensive involvement in safeguarding genomic stability, RPA has been shown to tightly associate with carcinogenesis. An early study aiming to identify autoantigens in breast cancer patients showed that antibodies against RPA32 can be detected prior to diagnosis [22]. In hepatocellular carcinoma and gastric cancer, higher RPA14 expression was found to correlate with a poor outcome [23,24]. Another study in bladder cancer showed that while in early stage low RPA32 levels positively correlated with patient outcome, at later stages in invasive tumors, both high RPA70 and RPA32 expression informed adverse prognosis [25]. Our study shows that high expression of all three RPA subunits, RPA70, RPA32 and RPA14, positively correlates with WHO malignancy stage and informs poor glioma patient survival.

Radiation therapy, which represents a standard-of-care for GBM patients, aims to eradicate cancer cells by inducing a broad viariety of DNA lesions, among those DSBs being reportedly the most detrimental in regards to maintaining genomic stability [20]. Here, we sought to interrogate the role of RPA in the radio-resistance of GBM and GSCs. We show that RPA expression is higher in GSCs than in DGCs. This is expected as our previous work has shown that GSCs are highly proliferative and exhibit greater ROS production compared to DGCs, which translates in higher number of ssDNA breaks and oxidative DNA lesions [15,19]. Because the key feature that distinguished tumor cells from most normal cells is their sustained proliferation, DNA replication has been harnessed as a semi-selective target in cancer therapies for decades [1,2,20]. Prior to the discovery of RPA inhibitors, inhibitors of RPA-ssDNA interaction have been shown to synergize with cisplatin, displaying potential for its therapeutic targeting [18]. The HAMNO inhibitor prevents RPA70’s interaction with ATR and so inhibits the activation of DDR in response to RS. The inhibitory effect of HAMNO was reportedly enhanced when combined with etoposide, a topoisomerase 1A inhibitor that induces RS [18]. Since gliomas and GSCs have been reported to undergo a constitutive RS, we speculated that HAMNO-mediated inhibition of RPA in GSCs would render this cell population sensitive to radiotherapy. Indeed, our study provides the evidence that RPA inhibition significantly impairs maintenance and survival of GSCs as monotherapy, an effect that is further potentiate by irradiation. The dose-dependent induction of DSBs after HAMNO treatment drives the GSCs into apoptosis.

Bélanger et al. (2018) reported that RPA exhaustion represents a major determinant of cisplatin sensitivity in ovarian cancer cell lines, a finding that harbors an important implication toward improving therapy of various cancers that initially respond to platinum-based agents but later relapse due to intrinsic or acquired drug resistance [26]. Wang et al. (2018) showed that RPA70 promotes tumor proliferation via CDK4/Cyclin-D pathway [27]. Toledo et al. (2013) showed that RPA depletion in cells under RS conditions triggers the activation of irreversible cell-cycle arrest and suggested that RPA-exhaustion-associated mitotic catastrophe might be a promising target in future cancer therapies [28]. In our model, targeting RPA reduces the proliferative index and prevents entry into mitosis, which is concordant with the studies mentioned above.

In cancer treatment, radiation therapy is second only to surgery in terms of its curative potential [12]. However, many cancers including GBM exhibit evasive radio-resistance, stressing the urgent need for identification and validation of putative radio-sensitizers. Here, we uncovered a novel role for RPA in the maintenance and radio-resistance of GSCs, thereby supporting its potential clinical significance as a biomarker in GBM.

## 4. Methodology

### 4.1. Tumor Dissociation, Cell Culture and MACS Sorting

GBM primary cells numbering 4121 in total were a generous gift of Prof. J.N. Rich (UCSD, CA, USA). Primary GBM cell lines (G01, G06, G40, G07, G16, G20; see Table S3) were derived from GBM (WHO grade IV glioma) tissue biopsies obtained at surgery after signing an informed consent as outlined by the Regional Danish Ethical Committee/Danish Data Protection Agency (protocol no.: H-3-2009-136_63114). Primary GBM cell lines were maintained as subcutaneous patient-derived xenografts by direct subcutaneous (SC) implantation of tissue biopsy chunks into the flanks of immunocompromised NOD.Cg-Prkdc^scid^ (Taconic Biosciences, Inc. Rensselaer, New York, NY, USA, cat.no. NOG-F). This protocol was approved by the Danish Welfare Law on Animal Experiments Act no 1306 (protocol no.: 2012-15-2934-00636/2018-15-0201-01391).

For in vitro experiments, resected SC tumors were dissociated using a Papain Dissociation Kit according to manufacturer’s instructions (Worthington Biochemical Corporation, Lakewood, New Jersey, USA cat.no. LK003150) and maintained as neurosphere cultures in neurobasal–A medium (Invitrogen, Carlsbad, CA, USA, cat.no. 12349-015,) supplemented with B27 minus Vitamin A (Invitrogen, Carlsbad, CA, USA, cat.no. 12587-010), Epidermal Growth Factor (EGF) (20 ng/mL) (R&D systems, Minneapolis, MN, USA, cat.no 236-EG-01M), Fibroblast Growth Factor (FGF) (20 ng/mL) (R&D systems, Minneapolis, MN, USA, cat.no. 4114-TC-01M), GlutaMax (Invitrogen, Carlsbad, CA, USA, cat.no. 35050-038) and antibiotics (Invitrogen, Carlsbad, CA, USA, cat.no. 15140-122). Cells were allowed to recover for 24 h prior their use for downstream experiments. Matched GBM cancer stem-like cells (GSCs; CD133+) and their differentiated counterpart populations (DGCs; CD133-) were isolated by magnetic (MACS) sorting using a CD133 microbeads kit (Miltenyi Biotec, Bergisch Gladbach, Germany, cat.no. 130-100-857). The GSCs were maintained in neurobasal–A medium, whilst the DGCs were maintained as monolayer in Dulbecco’s Modified Eagle Medium (DMEM) (Invitrogen, Carlsbad, CA, USA, cat.no. 31966-021), supplemented with 10% Fetal Bovine Serum (FBS) (Invitrogen, Carlsbad, CA, USA, cat.no. 26140095) and antibiotics (Invitrogen, Carlsbad, CA, USA, cat.no. 15140-122) as reported previously [14]. Both populations were validated functionally by sphere forming capacity and the expression of stem cell markers by immunoblotting for expression of SOX2 and GFAP.

HEK293T cells obtained from American Type Culture Collection (ATCC) were maintained a monolayer in DMEM (Invitrogen, Carlsbad, CA, USA, cat.no. 31966-021), supplemented with 10% FBS (Invitrogen, Carlsbad, CA, USA, cat.no. 26140095) and antibiotics (Invitrogen, Carlsbad, CA, USA, cat.no. 15140-122). For microscopy-based studies, cells were seeded on Geltrex (Invitrogen, Carlsbad, CA, USA, cat.no. 14113-202) coated plates.

Normal human astrocytes (NHA33, NHA26) obtained from ATCC were maintained in a monolayer in Astrocyte Medium (ScienCell Research Laboratories, Carlsbad, CA, USA, cat.no. SC-1801).

### 4.2. Flow Cytometry Analysis of Apoptosis

To measure early apoptosis, single cells were incubated with annexin V-Fluorescein (FITC) (dead cell apoptosis kit; BD biosciences, Franklin Lakes, NJ, USA, cat.no. V13242,) and processed according to the manufacturer’s instructions. For staining of cleaved caspase-3, cells were fixed for 30 min in ice-cold 96% ethanol, permeabilized using 0.25% Triton-X (Sigma-Aldrich, St. Louis, MI, USA, cat.no. T9284) and stained for 2 h with cleaved caspase-3 alexa flour 488 conjugated antibody (Cell Signaling, Danvers, Massachusetts, cat.no. 9669S,). All FACS flow cytometry samples were run on BD’s FACS Verse (BD biosciences, Franklin Lakes, NJ, USA) and analyzed using the FlowJo software version 10 (BD biosciences, Franklin Lakes, NJ, USA).

### 4.3. Drug Preparation and Irradiation

HAMNO was obtained from Sigma-Aldrich (Sigma-Aldrich, St. Louis, MI, USA, Cat.no. SML1234-5MG). Drug stock solutions were made in Dimethyl sulfoxide (DMSO) (Sigma-Aldrich, St. Louis, MI, USA, cat.no. D8418) at 30 μM and stored at −80 °C in dark and diluted fresh in the culture medium immediately before use.

For IR studies, cells were seeded on Geltrex (Invitrogen, Carlsbad, CA, USA, cat.no. 14113-202) coated plates in 96- or six-well format and induced with DSBs using YXLON smart (YXLON International A/S, Hamburg, Germany) at a rate of 0.04 Gy/s.

### 4.4. Proliferation Assay

For real-time analysis of cell proliferation over several days, the IncuCyte Zoom System (Essen BioScience Inc., Ann Arbor, MI, USA) was utilized. In brief, cells were seeded at a starting density of 3500 cells/well. After overnight recovery, cells were treated with appropriate doses of HAMNO and IR before incubation in the IncuCyte Zoom Live cell analysis system. Cell images were collected from four separate positions every four hours. For analysis, well confluency was exported.

### 4.5. Lentiviral Particle Preparation

The preparation of lentiviral particles and viral transduction was performed as described previously [1,2,29]. In brief, HEK293T cells were co-transfected with second generation packaging plasmids VSV and PAX and pLKO plasmids (RPA70 (Sigma-Aldrich, St. Louis, MI, USA, TRCN0000010983/TRCN00000318752), RPA32 (Sigma-Aldrich, St. Louis, MI, USA, TRCN00000231920) and RPA14 (Sigma-Aldrich, St. Louis, MI, USA, TRCN0000018861) using a calcium phosphate transfection kit (Takara Bio USA, Inc. Mountain View, CA, USA, cat.no. 631312). All viral particles were concentrated using PolyEthylene Glycol (PEG)-it virus precipitation solution (SBI, System Biosciences, Palo Alto, CA, USA, cat.no. LV810A-1) and stored in aliquots at −80 °C for later use.

### 4.6. Cell Viability

For cell viability dissociated cells were seeded at a density of 3500 cells/well in 100 ul of media in 96-well plates. Cell viability was measured at indicated time points using the CellTiter-Glo luminescent cell viability assay (Promega, Madison, WI, USA, cat.no. G7571,) according to the manufacturer’s instructions or assessed using the IncuCyte Life cell analysis system (EssenBioscience Inc., Ann Arbor, MI, USA). Results were analysed using Microsoft Excel and GraphPad Prism software version 8 (GraphPad Software, San Diego, CA, USA).

### 4.7. Neurosphere Formation and Extreme Limiting Dilution Assay (ELDA)

For neurosphere formation single cells were plated at a density of 2000 cells/mL in 2ml of complete media in six-well plates. After 7–10 days, plates were observed for sphere formation. For ELDA cells were seeded at a density of 25, 50, 250, 500 and 750 cells per well in 96-wells. The frequency of neurosphere formation (surrogated for self-renewal) was evaluated 10 days after exposure to HAMNO or transduction with shRNA targeting RPA70. Wells were scored positive or negative for the presence of at least one neurosphere. The estimated stem cell frequency was calculated using extreme limiting dilution analysis as reported previously [30].

### 4.8. Immunoblotting

Protein extracts were prepared using whole lysis buffer (WLB; 50 mM Tris–HCl (Trizma Base; Sigma-Aldrich, St. Louis, MI, USA, cat.no. 93350. HCl; Sigma-Aldrich, St. Louis, MI, USA, cat.no. H1758), 10% Glycerol (Sigma-Aldrich, St. Louis, MI, USA, cat.no. G5516), 2% SDS (Bio-Rad Laboratories, Inc., Hercules, CA, USA, cat.no. 161-0418-MSDS) and water) and protein concentrations were determined by Pierce BCA Protein Assay (Thermo Fisher Scientific, Waltham, MA, USA, cat.no. 23227,). Cell lysates (25–30 µg) were separated by electrophoresis on SDS-PAGE gels (Bio-Rad Laboratories, Inc., Hercules, CA, USA). Proteins were transferred onto nitrocellulose membranes using the trans-blot turbo transfer system (Bio-Rad Laboratories, Inc., Hercules, CA, USA) and incubated with appropriate primary antibodies: RPA14 (Abcam, Cambridge, UK, cat.no. ab97436,); RPA32 (Abcam, Cambridge, UK, cat.no. ab2175); RPA70 (Abcam, Cambridge, UK, cat.no. ab79398); SOX2 (Merck Millipore, Burlington, MA, USA, cat.no. MAB4343); Glial Fibrillary Acidic Protein (GFAP) (Agilent Dako, Santa Clara, CA, USA, cat.no. Z0334); α-Tubulin (Sigma-Aldrich, St. Louis, MI, USA, cat.no. T9026-0.2ML); GAPDH (GeneTex, Irvine, CA, USA, cat.no. GT239); β-actin (Santa Cruz Biotechnologies, Dallas, TX, USA, cat.no. SC-47778) and species-specific secondary antibodies: HRP goat anti-rabbit IgG antibody (Vector Laboratories Inc, Burlingame, CA, USA, cat.no. PI-1000,) and HRP horse anti-mouse IgG antibody (Vector Laboratories Inc, Burlingame, CA, USA, cat.no. PI-2000). Amersham ECL prime western blotting detection reagent (GE Healthcare, Chicago, IL, USA, cat.no. RPN2232,) was used for detection using Image Lab software version 5.0 (Bio-Rad Laboratories, Inc., Hercules, CA, USA).

### 4.9. Immunofluorescence and Image Analysis

For immunofluorescence staining, cells grown on geltrex-coated coverslips (Invitrogen, Carlsbad, CA, USA, cat.no. 14113-202) and fixed with 4% paraformaldehyde (PFA) (Sigma-Aldrich, St. Louis, MI, USA, cat.no. P6148), permeabilized using 0.25% Triton-X (Sigma-Aldrich, St. Louis, MI, USA, cat.no. T9284) followed by blocking for 30 min in 10% Bovine Serum Albumin (BSA) (Bovine Albumin Fraction V, 7.5% solution; Thermo Fisher Scientific, Waltham, MA, USA, cat.no. 15260037). Cells were incubated with respective primary antibodies (H2AX (Ser139) (Merck Millipore, Burlington, MA, USA, cat.no. 05-636); Cyclin A (H-432) (Santa Cruz Biotechnologies, Dallas, TX, USA, cat.no. sc-751) in 2% BSA (Bovine Albumin Fraction V, 7.5% solution; Thermo Fisher Scientific, Waltham, Massachusetts, cat.no. 15260037) for 2 h followed by incubation with appropriate secondary conjugated antibodies (Alexa Flour 488 (Invitrogen, Carlsbad, CA, USA, cat.no. A11029); Alexa Flour 568 (Invitrogen, Carlsbad, CA, USA, cat.no. A11036). Nuclei were counterstained with 4,6-Diamidino-2-Phenylindole (DAPI) (Sigma-Aldrich, St. Louis, MI, USA, cat.no. D9542-10MG). Automated imaging and analysis were performed using Olympus Scan-R screening station equipped with Scan-R analysis software as described previously [29].

### 4.10. In Silico Analysis of Public Data Sets

The expression of *RPA70*, *RPA32* and *RPA14* mRNA in normal brain, malignant gliomas (WHO grade II III and IV) and its correlation patient survival (Kaplan-Meier survival analysis) was performed using publically available microarray expression data sets (REMBRANDT, TCGA_GBM and TCGA_GBMLGG) via the gliovios platform: http://gliovis.bioinfo.cnio.es/ [14]. The multivariate Cox proportional hazard analysis was performed using the coxph() function from the “survival” R package using log2 transformed microarray expression data [31]. For the coxph regression the REMBRANDT data were trimmed to exclude patients where histology or WHO grade are not available.

### 4.11. Statistical Analysis

All statistical analyses were performed using GraphPad Prism 7 software or R 3.6.1. Statistical significance was assessed by the unpaired t-test or two-way ANOVA as indicated. Precise details on statistical tests can be found in the figure legends. *p*-values <0.05 were considered to be significant.

## Supplementary Materials

The following are available online at https://www.mdpi.com/1422-0067/21/5/1588/s1; Figure S1. Figure providing supplementary information to main Figure 1. Figure S2. Figure providing supplementary information to main Figure 1. Table S1. A multi-variate Cox proportional hazard analysis—REMBRANDT data set. Table S2. A multi-variate Cox proportional hazard analysis—TCGA data sets. Table S3. Primary GBM cell lines characteristics.
