# Supplementary material for: Replication Protein A (RPA) Mediates Radio-Resistance of Glioblastoma Cancer Stem-Like Cells
Source: Int J Mol Sci. 2020 Feb 26;21(5):1588. doi: 10.3390/ijms21051588 (PMC7084771; doi:10.3390/ijms21051588)
Supplement: Supplementary file 1 [file ijms-21-01588-s001.pdf]

International Journal of
Molecular Sciences

MDPI

SUPPLEMENTARY FIGURES AND FIGURE LEGENDS

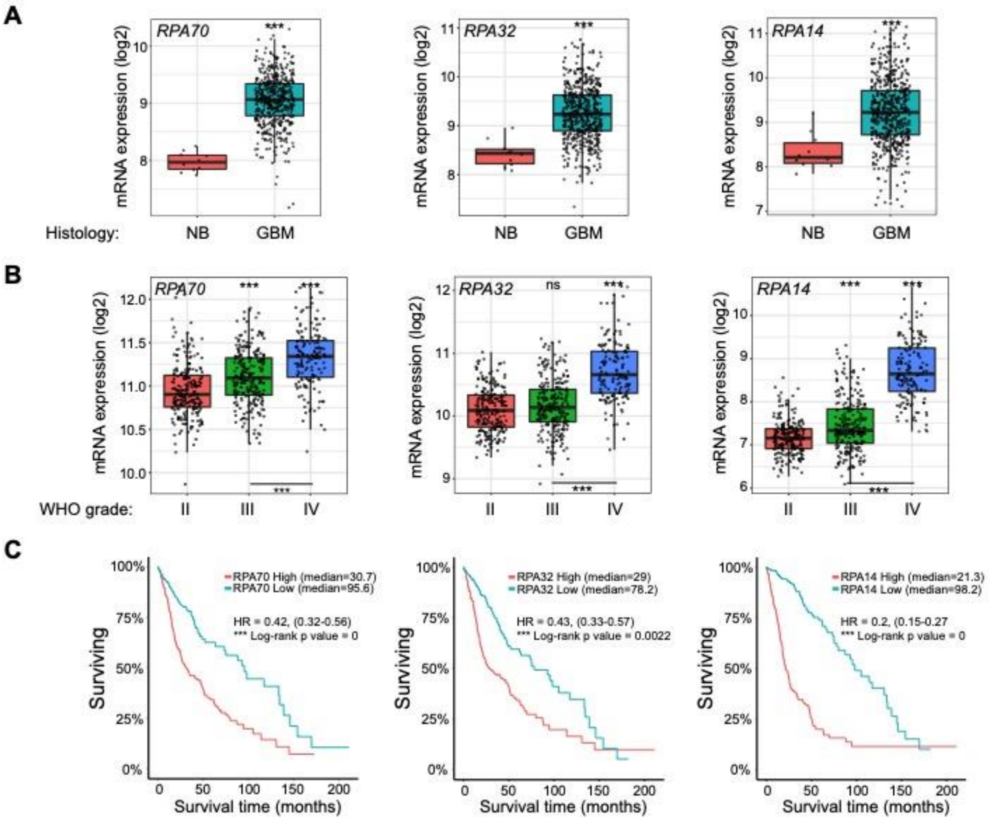

Supplementary Figure S1. Figure providing supplementary information to main Figure 1.
(A) *RPA70*, *RPA32*, *RPA14* expression analysis of TCGA data (The Cancer Genome Atlas GBMLGG cohort) in GBM and normal brain (NB) controls. **(B)** *RPA70*, *RPA32*, *RPA14* expression analysis of TCGA glioma data set comparing WHO grade II, III and IV gliomas. Data are presented as mean ± SD. Statistical significance was tested using Tukey's Honestly Significant Difference test, HSD. ns: not significant; *p<0.05; **p<0.01. **(C)** Kaplan-Meier survival analysis of TCGA glioma data set shows that high RPA expression (all subunits) informs poor patient prognosis.

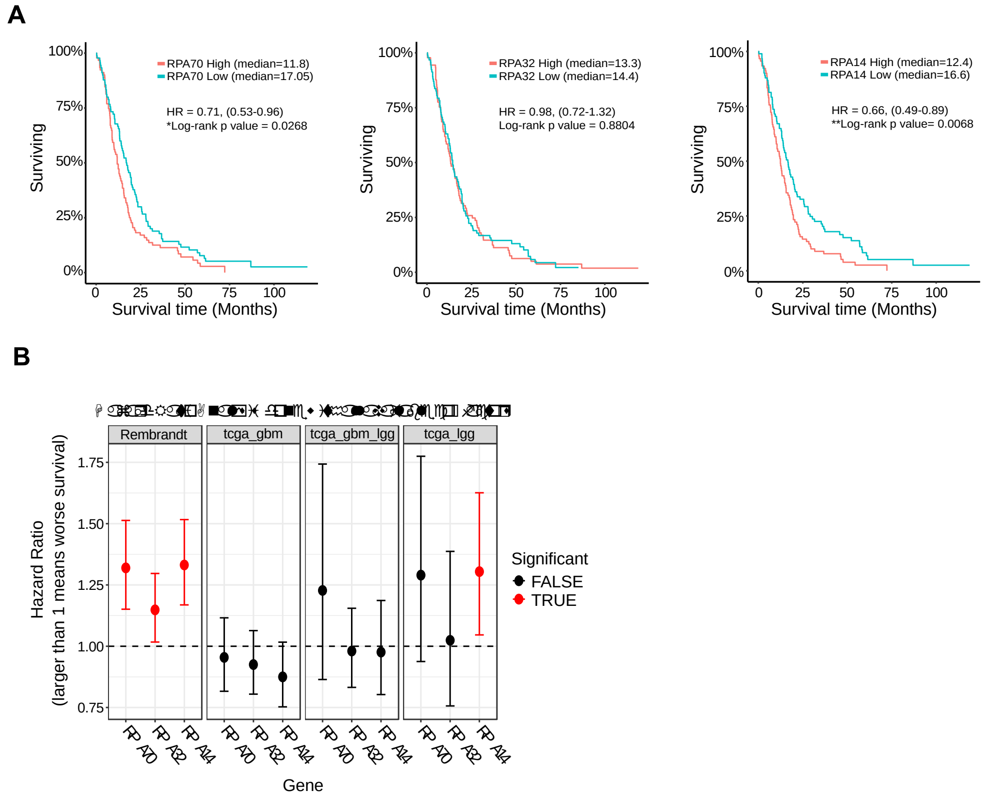

Supplementary Figure S2. Figure providing supplementary information to main Figure 1.
(A) Kaplan-Meier survival analysis of REMBRANDT GBM data set shows that high RPA expression (RPA70 and RPA14 subunits) informs poor patient prognosis. **(B)** An overview of available co-variates and their correlation with RPA subunits in regards to informing patients' survival in REMBRANT and TCGA data sets.

Supplementary Table S1. A multi-variate Cox proportional hazard analysis – REMBRANDT data set.

Data	Clinical cofactor	Hazard ratio	Hazard ratio 95% confidence interval	p_value
Rembrandt	Grade_IV_GBM	2,761	1.794 - 4.25	3,87541E-06
Rembrandt	RPA3_expression	1,247	1.059 - 1.47	0,007947761
Rembrandt	RPA1_expression	1,214	1.029 - 1.43	0,021534727
Rembrandt	ATRX_expression	1,086	0.963 - 1.22	0,180268743
Rembrandt	Grade_III_Oligodendroglioma	1,464	0.803 - 2.67	0,213156129
Rembrandt	Grade_III_Astrocytoma	1,360	0.822 - 2.25	0,231625911
Rembrandt	RPA2_expression	0,950	0.819 - 1.10	0,493812318
Rembrandt	Grade_II_Oligodendroglioma	1,174	0.606 - 2.27	0,634352115
Rembrandt	gender_male	NA	NA	NA
Rembrandt	IDH1_status_Wild_type	NA	NA	NA
Rembrandt	MGMT_status_Unmethylated	NA	NA	NA
Rembrandt	Age	NA	NA	NA

Supplementary Table S2. A multi-variate Cox proportional hazard analysis – TCGA data sets.

Data	Clinical cofactor	Hazard ratio	Hazard ratio 95% confidence interval	p_value
tcga_gbm	Age	1,028	1.015 - 1.04	1,4538E-05
tcga_gbm	gender_male	1,660	1.228 - 2.24	0,000984265
tcga_gbm	IDH1_status_Wild_type	2,455	1.132 - 5.32	0,022900353
tcga_gbm	MGMT_status_Unmethylated	1,256	0.948 - 1.66	0,11285813
tcga_gbm	RPA3_expression	0,883	0.739 - 1.05	0,170738562
tcga_gbm	ATRX_expression	1,091	0.919 - 1.30	0,319268788
tcga_gbm	RPA2_expression	0,987	0.824 - 1.18	0,883562296
tcga_gbm	RPA1_expression	0,993	0.835 - 1.18	0,94060235
tcga_gbm_lgg	IDH1_status_Wild_type	3,943	2.492 - 6.24	4,55379E-09
tcga_gbm_lgg	Grade_IV_GBM	20,489	3.504 -119.80	0,000803303
tcga_gbm_lgg	gender_male	1,524	1.185 - 1.96	0,001007619
tcga_gbm_lgg	Grade_III_Astrocytoma	3,974	0.937 - 16.86	0,061339739
tcga_gbm_lgg	ATRX_expression	1,340	0.902 - 1.99	0,146856271
tcga_gbm_lgg	Grade_III_Oligoastrocytoma	2,922	0.634 - 13.48	0,169186315
tcga_gbm_lgg	RPA1_expression	1,306	0.890 - 1.92	0,173071362
tcga_gbm_lgg	Grade_III_Oligodendroglioma	1,594	0.349 - 7.29	0,547461188
tcga_gbm_lgg	RPA2_expression	0,941	0.770 - 1.15	0,549288399
tcga_gbm_lgg	Grade_II_Oligoastrocytoma	1,442	0.295 - 7.05	0,651138341
tcga_gbm_lgg	Grade_II_Oligodendroglioma	0,761	0.161 - 3.59	0,730545829
tcga_gbm_lgg	RPA3_expression	0,976	0.780 - 1.22	0,833431443
tcga_gbm_lgg	MGMT_status_Unmethylated	NA	NA	NA
tcga_gbm_lgg	Age	NA	NA	NA
tcga_lgg	IDH1_status_Wild_type	3,749	2.007 - 7.00	3,40239E-05
tcga_lgg	RPA3_expression	1,389	1.071 - 1.80	0,013380746
tcga_lgg	Grade_III_Astrocytoma	3,638	0.835 - 15.85	0,085434123
tcga_lgg	RPA2_expression	0,761	0.535 - 1.08	0,129149523
tcga_lgg	Grade_III_Oligoastrocytoma	3,056	0.621 - 15.04	0,169456674
tcga_lgg	RPA1_expression	1,281	0.899 - 1.82	0,169943065
tcga_lgg	Grade_III_Oligodendroglioma	1,785	0.362 - 8.81	0,476475099
tcga_lgg	gender_male	0,867	0.520 - 1.45	0,584857639
tcga_lgg	Grade_II_Oligoastrocytoma	1,476	0.292 - 7.45	0,637657112
tcga_lgg	Grade_II_Oligodendroglioma	0,707	0.142 - 3.52	0,671995752
tcga_lgg	ATRX_expression	0,978	0.692 - 1.38	0,900530695
tcga_lgg	MGMT_status_Unmethylated	NA	NA	NA
tcga_lgg	Age	NA	NA	NA

Supplementary Table S3. Primary GBM cell lines characteristics.

Primary GBM cell line	Passage	Diagnosis	Age at diagnosis	Gender	Survival (Months)	Primary /Recurrent	MGMT	IDH1	ATRX
**4121**	N.A.	WHO gr. IV glioma/GBM	N.A.	N.A.	N.A.	N.A.	N.A.	N.A.	N.A.
**G01**	xp8	WHO gr. IV glioma/GBM	41	M	†11	Primary	N.A.	N.A.	N.A.
**G06**	xp4	WHO gr. IV glioma/GBM	80	M	†0	Primary	Non-methylated	WT	N.A.
**G40**	xp2	WHO gr. IV glioma/GBM	62	M	†11	Recurrent	N.A.	WT	WT
**G07**	xp3	WHO gr. IV glioma/GBM	54	M	†17	Recurrent	N.A.	N.A.	N.A.
**G16**	xp2	WHO gr. IV glioma/GBM	76	M	†45	Primary	Methylated	WT	N.A.
**G20**	xp1	WHO gr. IV glioma/GBM	40	M	†16	Primary	Non-methylated	WT	WT
